# Active Case Finding of Chronic Obstructive Pulmonary Disease in Romanian Primary Care

**DOI:** 10.3390/medicina62091820

**Published:** 2026-09-21

**Authors:** Andreea Narcisa Iana, Daniela Gurgus, Roxana Folescu

**Affiliations:** Family Clinic, Center of Preventive Medicine, University of Medicine and Pharmacy Victor Babes, 300023 Timișoara, Romania; gurgus.daniela@umft.ro (D.G.); folescu.roxana@umft.ro (R.F.)

**Keywords:** chronic obstructive pulmonary disease, primary care, CAT score, mMRC scale

## Abstract

*Background and Objectives*: Chronic obstructive pulmonary disease (COPD) is one of the leading causes of death worldwide and represents a major contributor to the global burden of disease. According to the World Health Organization it is the third leading cause of death worldwide and the seventh leading cause of poor health worldwide. Our aim was to evaluate the effectiveness of active case finding of COPD in Romanian primary care through the COPD Assessment Test (CAT) and the Modified Medical Research Council (mMRC) dyspnea scale and post-bronchodilator (post-BD) spirometry. *Materials and Methods*: A prospective active case-finding study was conducted among 220 proactively selected patients with an age over 40 years and a documented history of at least one major respiratory risk factor. The study enrolled patients from one General Practitioner (GP) office. The exclusion criteria were malignancies and previously diagnosed obstructive disease. Demographics, tobacco exposure, clinical history and validated case-finding questionnaires (CAT and mMRC) were recorded. All participants underwent diagnostic post-bronchodilator spirometry. Airflow limitation was defined as a post-bronchodilator FEV_1_/FVC <0.70. The statistical analysis was performed with The Statistical Package for the Social Sciences version 27 (SPSS, Chicago, IL, USA). *Results*: Spirometry confirmed a positive COPD diagnosis in 48.2% *(n* = 106) of the study cohort. Severity staging revealed that 34.9% met criteria for GOLD 1 (Mild), 49.1% for GOLD 2 (Moderate), 15.1% for GOLD 3 (Severe), and 0.9% for GOLD 4 (Very Severe). Smoking history (*p* < 0.001) was significantly higher in the COPD group. While the mMRC scale at a cut-off ≥2 demonstrated excellent diagnostic specificity (82.5%), its low sensitivity (41.5%) means that relying solely on breathlessness in primary care questionnaires would result in missing probably more than half of the patients with a positive diagnosis. In contrast, the CAT score at a threshold ≥17 offers a more balanced trade-off (64.2% sensitivity, 74.6% specificity) for active case finding of COPD patients. *Discussion*: The findings of this study acknowledge the clinical utility of using validated questionnaires in active case finding of COPD in Romanian primary care. A comprehensive evaluation of symptoms—incorporating cough, sputum production, and sleep impact—provides a much more reliable clinical trigger for diagnostic spirometry than dyspnea alone. While tobacco smoking remains the predominant driver of airflow limitation in our cohort, the identification of obstruction in 4.7% of never-smokers acknowledges the clinical necessity of accounting for non-smoking risk factors, occupational dust, environmental exposures and documented histories of recurrent winter bronchitis, persistent morning cough, or unexplained sputum production. In the study cohort, a CAT score ≥17 showed the best observed balance between sensitivity and specificity; this threshold should be considered cohort-specific and requires external validation before being adopted as a universal screening cut-off. This study had limitations, including the small, single-center sample size, which may limit generalization to the Romanian population. *Conclusions*: The results support the implementation of a targeted active case-finding strategy in Romanian primary care. These findings support further evaluation of targeted case-finding strategies incorporating symptom burden and smoking exposure as well as algorithms like spirometry on individuals over 40 who present with a CAT score ≥ 17, or a tobacco history.

## 1. Introduction

Chronic obstructive pulmonary disease (COPD) is one of the leading causes of death worldwide and represents a major contributor to the global burden of disease. According to the World Health Organization it is the third leading cause of death worldwide, causing 3.4 million deaths in 2023, approximately 6% of all global deaths, and COPD is the seventh leading cause of poor health worldwide; tobacco smoking accounts for over 70% of COPD cases in high-income countries, as published in 2025 [1].

In Romania, the prevalence of COPD has been estimated at 9.3% among individuals over 40 years old by the Romanian Society of Pneumology in an abstract published in 2012 [2] but more probably remain undiagnosed.

In clinical practice, diagnosis is usually based on symptoms of breathlessness, cough and sputum, a history of smoking, or exposure to other noxious agents and airflow limitation diagnosed by spirometry that is not fully reversible post-BD, with FEV_1_/FVC < 0.7 [3].

Given the central role of primary care in Romanian healthcare and their longitudinal relationships with patients and families, it can offer a critical opportunity to strengthen COPD active case finding and start early interventions.

By focusing on a real-world primary care environment, this study seeks to explore the practical integration of active case finding of COPD comparing the diagnostic utility in Romanian primary care of validated questionnaires such as the CAT and mMRC dyspnea scale and post-BD spirometry and to provide important preliminary data that may inform future large scale investigations.

Patients with a CAT score ≥10 [4] or an mMRC score ≥2 represent more prominent symptoms. The CAT is a standardized questionnaire that quantifies the quality of life of a COPD patient. It consists of eight items: cough, sputum, chest tightness, breathlessness, activity limitations, confidence leaving home, sleep quality and energy levels. Each is scored on a six point scale ranging from 0 to 5. The overall score ranges from 0 to 40, where higher scores indicate a more severe impact on the patient. CAT score interpretation is 0–10: low clinical impact, 11–20: medium clinical impact, 21–30: high clinical impact, 31–40: very high clinical impact. The mMRC score ranges from 0 to 4 and quantifies the breathlessness of the patients; an mMRC score ≥2 indicates a higher symptom burden for patients [3].

The literature notes that, despite the potential benefits of COPD early detection, several challenges remain in this area, like the lack of spirometry use and the failure to recognize early signs of COPD [5].

Individuals with undiagnosed COPD had worse health-related quality of life compared to those without airflow obstruction [6]. Furthermore, compared to early diagnosis, late detection of COPD is related to higher exacerbation rate and increased comorbidities and costs [7,8].

So, evaluating current practices, barriers and feasible strategies for a systematic early diagnosis of COPD in primary care is essential.

## 2. Materials and Methods

### Study Design and Participants

This study took place in a General Practitioner Office in Timisoara, Romania, from 12 January to 30 June 2026. The study was implemented in compliance with the rules of good clinical practice; the protocol approval no. is 5/8 January 2026. All participants provided their written consent before joining the study.

The prospective cross-sectional active case-finding study was conducted among 220 consecutive patients with an age over 40 years. Patients were proactively selected and invited to participate if they met the primary inclusion criterion of age > 40 years combined with a documented history of at least one major respiratory risk factor including: a substantial history of tobacco exposure (>20 pack-years), prolonged exposure to non-smoking risk factors, documented histories of recurrent winter bronchitis, persistent morning cough, or unexplained sputum production recorded in the clinic’s electronic health records within the previous 24 months. The exclusion criteria were malignancies and previously diagnosed obstructive disease. Demographics, clinical history, tobacco exposure, and validated case-finding questionnaires (CAT and mMRC) were recorded for all patients.

All participants underwent diagnostic post-bronchodilator spirometry. Spirometry was performed with Contec Spirometry SP80B (Contec Medical Systems Co., Ltd., New Delhi, India). To ensure absolute methodological validity, strict adherence to the ATS/ERS 2019 standardization guidelines was maintained. Equipment calibration and volume verification were performed daily. We used the post-bronchodilator COPD protocol: pre-BD patients performed ≥3 acceptable blows, with good coaching; then they were given, according to the local protocol, salbutamol 400 µg; and post-BD the patients repeated ≥3 acceptable blows, after a strict waiting time interval of 15 min. Airflow limitation was defined as post-bronchodilator FEV_1_/FVC < 0.70 which supported airflow obstruction consistent with COPD. Predicted normal values and spirometric percentiles were calculated using the Global Lung Function Initiative (GLI-2012) reference equations.

The statistical analysis was performed with The Statistical Package for the Social Sciences version 27 (SPSS, Chicago, IL, USA).

The statistical results are presented as the mean ± standard deviation/standard error, percentages, or median (minimum–maximum). The primary statistical comparisons were evaluated using an alpha of 0.05, with a *p*-value < 0.001 considered highly significant. We utilized receiver operating characteristic (ROC) curve analysis and the Chi-square test.

Patients were stratified into two clinical cohorts based on the presence of objective airflow limitation (post-BD FEV1/FVC < 0.70). Independent two-sample *t*-tests were used to compare means between the non-COPD and COPD groups for normally distributed variables (age). The Mann–Whitney U test was used for non-parametric continuous scores (CAT score, mMRC scale, pack-years). Categorical associations—such as smoking status and gender distributions—were evaluated using Pearson’s Chi-square (χ^2^) test.

## 3. Results

Spirometry confirmed a positive COPD diagnosis in 48.2% (*n* = 106) of the study cohort (Table 1). Severity staging revealed that 34.9% (*n* = 37) met criteria for GOLD 1 (Mild), 49.1% (*n* = 52) for GOLD 2 (Moderate), 15.1% *(n* = 16) for GOLD 3 (Severe), and 0.9% (*n* = 1) for GOLD 4 (Very Severe). Smoking history (*p* < 0.001) was significantly higher in the COPD group.

Tobacco history increased the likelihood of positive COPD spirometry (Table 2). Active or past smoking history was present in 95.3% of all spirometry-confirmed cases (*p* < 0.001). In total, 4.7% of the patients with persistent airflow obstruction were never-smokers, demonstrating the presence of non-smoking COPD risk factors in this primary care population.

In our cohort of 220 patients, the CAT score significantly outperformed the mMRC scale, as demonstrated by the distinct gap in their areas under the receiver operating characteristic curve (AUC: 0.753 vs. 0.654, *p* = 0.004) (Table 3).

While the mMRC scale at a cut-off ≥2 demonstrated excellent diagnostic specificity (82.5%), its low sensitivity (41.5%) means that relying solely on breathlessness in a family medicine setting would result in missing more than half of the COPD patients. In contrast, the CAT score at a threshold ≥17 offers a more balanced trade-off (64.2% sensitivity, 74.6% specificity) (Table 3).

## 4. Discussion

The findings of our study acknowledge the clinical utility of using validated questionnaires and post-BD spirometry, as well as their benefit in active case finding of COPD in the general Romanian population.

A comprehensive evaluation of symptoms—incorporating cough, sputum production, and sleep impact—provides a much more reliable clinical trigger for diagnostic spirometry than dyspnea alone.

Our data emphasized higher case finding in elderly patients, confirming that prevention and management should be integrated into targeted National Health Programs for elderly patients with symptoms or risk factors, and that they should be referred for spirometry [9].

Nearly half of diagnosed patients (49.1%) were diagnosed with COPD GOLD 2 and 34.9% were diagnosed with COPD GOLD 1; most of these patients are frequently asymptomatic and remain undiagnosed, but routine spirometry and evaluation can trigger comprehensive clinical assessments and early interventions.

While tobacco smoking remains the predominant driver of airflow limitation in our cohort, the identification of obstruction in 4.7% of never-smokers highlights the clinical necessity of accounting for non-smoking risk factors, occupational dust, environmental exposures and documented histories of recurrent winter bronchitis, persistent morning cough, or unexplained sputum production.

We have to note that single spirometry or clinical respiratory symptoms alone are not enough for accurate COPD diagnosis. A comprehensive approach including clinical assessment and follow-up spirometry should be taken into consideration for the diagnosis and management of COPD [10], so our study provides only preliminary data that may inform future large-scale investigations.

A narrative review in the European Journal of Internal Medicine in 2020 found that to reduce the significant burden of COPD, a concerted effort is needed from healthcare providers to drive early identification of those at risk, improve reporting and increase preventative care [11]; we need to focus on active case finding in primary care.

Our study supports the conclusion of the Romanian paper published in Pneumologia this year [12] that it is necessary to expand access to spirometry in primary care, including training and certification for family physicians.

Active case finding of COPD in primary care with accessible tools like spirometry and questionnaires like CAT and mMRC, leading to early diagnosis, will significantly improve patients’ quality of life and life expectancy in the long run.

This study proves that assessing comprehensive symptom burden provides significant predictive power beyond standard demographic and tobacco exposure details.

To address the prominent issue of undiagnosed airflow limitation in primary care, several validated, population-level screening questionnaires have been established to identify individuals at risk who require diagnostic spirometry; therefore, we have to mention other validated questionnaires like the COPD Population Screener (COPD-PS) [13], the Lung Function Questionnaire (LFQ) [14], the COPD Screening Questionnaire (COPD-SQ) [15], and the COPD Assessment in Primary Care to Identify Undiagnosed Respiratory Disease and Exacerbation Risk (CAPTURE) tool [16]. Our study focused on the eight-item CAT score, which studied patients completed, to quantify the impact of symptoms on their daily life.

The clinical value of early diagnosis depends on subsequent interventions, such as smoking cessation, vaccination, pulmonary rehabilitation, appropriate pharmacological therapy and/or exacerbation prevention.

Primary care may also support severe COPD management through repeated eosinophil assessment and referral to a pneumologist for biologic therapy [17] and further management.

Implementing a targeted active case-finding strategy within General Practitioner Offices would facilitate an early detection of COPD.

This study has some limitations, which we must note. The sample size (*n* = 220) was small and from a single primary care center. These factors introduce potential selection bias and limit the generalizability of the findings to the broader Romanian population, including rural populations and patients managed in other healthcare settings.

The limitations include the use of a fixed FEV_1_/FVC ratio in an older population, and the lack of external validation. In the study cohort, a CAT score ≥ 17 showed the best observed balance between sensitivity and specificity; this threshold should be considered cohort-specific and requires external validation before being adopted as a universal screening cut-off. According to GOLD guidance, a CAT score ≥ 10 identifies patients with more significant respiratory symptoms and is commonly used for symptom assessment; it is not, by itself, a diagnostic threshold for COPD. In the present study, a CAT score ≥ 17 was selected as a study-specific ROC-derived threshold rather than as a replacement for the GOLD symptom threshold. A CAT score ≥ 17 should be interpreted as a preliminary, cohort-specific screening threshold requiring external validation rather than as a guideline-mandated cut-off, its use should be constrained to preliminary risk-stratification rather than a standard diagnostic tool.

Comorbidities and prior chronic medications were not systematically assessed. Conditions such as cardiovascular disease, obesity, asthma, or sleep-related disorders may influence respiratory symptoms and CAT/mMRC scores and could therefore affect screening performance. Medication use may also influence symptoms and spirometric measurements. Future multi-center studies should collect standardized comorbidity and medication data and prospectively validate the screening thresholds.

## 5. Conclusions

The results support the implementation of a targeted active case-finding strategy in Romanian primary care. In this single-center Romanian primary care cohort, the CAT score showed better discrimination for post-bronchodilator airflow obstruction than mMRC. Our findings support further prospective evaluation of targeted case-finding strategies incorporating respiratory symptom burden, smoking exposure and other environmental COPD risk factors, as well as algorithms like spirometry, on individuals over 40 who present with a CAT score ≥ 17 or a tobacco history.

## Figures and Tables

**Table 1:** Baseline characteristics.

Parameter	COPD Present (*n* = 106)	COPD Absent (*n* = 114)	Test Statistic	*p*-Value
Age (years), mean ± SD	66.4 ± 11.2	59.8 ± 10.9	t = −4.43	<0.001
Gender, *n* (%)			χ^2^ = 3.30	0.069
Male, *n* (%)	65 (61.3%)	56 (49.1%)		
Female, *n* (%)	41 (38.7%)	58 (50.9%)		
Smoking Status, *n* (%)			χ^2^ = 45.14	<0.001
Never Smoker	5 (4.7%)	50 (43.9%)		
Ex-Smoker	59 (55.7%)	40 (35.1%)		
Current Smoker	42 (39.6%)	24 (21.1%)		
Pack-Years, median (IQR)	31.8 (18.5–49.3)	5.1 (0.0–18.2)	U = 2104.5	<0.001
CAT Score, median (IQR)	19 (14–24)	10 (6–15)	U = 2985.0	<0.001
mMRC Score, median (IQR)	1.0 (1–2)	1.0 (0–1)	U = 4181.0	<0.001
Post-BD FEV1/FVC, mean ± SD	0.61 ± 0.06	0.78 ± 0.04	t = 24.81	<0.001

**Table 2:** Patients’ smoking status stratified by group.

Study Group	COPD Absent	COPD Present	Total Patients
Never Smoker	50	5	55
Smoker (Current/Ex)	64	101	165
Total Patients	114	106	220

**Table 3:** Comparative ROC curve CAT score and mMRC scale.

Metric	CAT Score (≥17)	mMRC Scale (≥2)
Area Under the Curve (AUC)	0.753 [0.690–0.816]	0.654 [0.581–0.727]
Asymptotic *p*-value	<0.001	<0.001
True Positives/False Negatives (*n*)	68/38	44/62
True Negatives/False Positives (*n*)	85/29	94/20
Sensitivity (95% CI)	64.2% [54.7–72.6%]	41.5% [32.6–51.0%]
Specificity (95% CI)	74.6% [65.9–81.7%]	82.5% [74.4–88.3%]
Youden Index (J)	0.39	0.24
Positive Likelihood Ratio (LR^+^)	2.53	2.37
Negative Likelihood Ratio (LR^−^)	0.48	0.71
PPV (Study Prevalence: 48.2%)	70.2%	68.8%
NPV (Study Prevalence: 48.2%)	69.1%	60.3%
PPV (Target Prevalence: 9.3%)	20.6%	19.5%
NPV (Target Prevalence: 9.3%)	95.3%	93.3%

Abbreviations: CI, confidence interval; LR, likelihood ratio; mMRC, Modified Medical Research Council dyspnea scale; NPV, negative predictive value; PPV, positive predictive value; ROC, receiver operating characteristic. Note: DeLong pairwise comparison of CAT vs. mMRC AUC values revealed a statistically significant difference (*p* = 0.004). Target prevalence (9.3%) represents the estimated baseline COPD prevalence in Romanian primary care.

## Data Availability

The anonymized participant-level dataset and the statistical output underlying the diagnostic accuracy analyses are available on request.
